# Multiscale Local Enhancement Deep Convolutional Networks for the Automated 3D Segmentation of Gross Tumor Volumes in Nasopharyngeal Carcinoma: A Multi-Institutional Dataset Study

**DOI:** 10.3389/fonc.2022.827991

**Published:** 2022-03-18

**Authors:** Geng Yang, Zhenhui Dai, Yiwen Zhang, Lin Zhu, Junwen Tan, Zefeiyun Chen, Bailin Zhang, Chunya Cai, Qiang He, Fei Li, Xuetao Wang, Wei Yang

**Affiliations:** ^1^ School of Biomedical Engineering, Southern Medical University, Guangzhou, China; ^2^ Guangdong Provincial Key Laboratory of Medical Image Processing, Southern Medical University, Guangzhou, China; ^3^ Department of Radiation Therapy, The Second Affiliated Hospital of Guangzhou University of Chinese Medicine, Guangzhou, China; ^4^ Department of Oncology, The Fourth Affiliated Hospital of Guangxi Medical University, Liuzhou, China

**Keywords:** nasopharyngeal carcinoma, segmentation, deep learning, radiotherapy, CT images

## Abstract

**Purpose:**

Accurate segmentation of gross target volume (GTV) from computed tomography (CT) images is a prerequisite in radiotherapy for nasopharyngeal carcinoma (NPC). However, this task is very challenging due to the low contrast at the boundary of the tumor and the great variety of sizes and morphologies of tumors between different stages. Meanwhile, the data source also seriously affect the results of segmentation. In this paper, we propose a novel three-dimensional (3D) automatic segmentation algorithm that adopts cascaded multiscale local enhancement of convolutional neural networks (CNNs) and conduct experiments on multi-institutional datasets to address the above problems.

**Materials and Methods:**

In this study, we retrospectively collected CT images of 257 NPC patients to test the performance of the proposed automatic segmentation model, and conducted experiments on two additional multi-institutional datasets. Our novel segmentation framework consists of three parts. First, the segmentation framework is based on a 3D Res-UNet backbone model that has excellent segmentation performance. Then, we adopt a multiscale dilated convolution block to enhance the receptive field and focus on the target area and boundary for segmentation improvement. Finally, a central localization cascade model for local enhancement is designed to concentrate on the GTV region for fine segmentation to improve the robustness. The Dice similarity coefficient (DSC), positive predictive value (PPV), sensitivity (SEN), average symmetric surface distance (ASSD) and 95% Hausdorff distance (HD95) are utilized as qualitative evaluation criteria to estimate the performance of our automated segmentation algorithm.

**Results:**

The experimental results show that compared with other state-of-the-art methods, our modified version 3D Res-UNet backbone has excellent performance and achieves the best results in terms of the quantitative metrics DSC, PPR, ASSD and HD95, which reached 74.49 ± 7.81%, 79.97 ± 13.90%, 1.49 ± 0.65 mm and 5.06 ± 3.30 mm, respectively. It should be noted that the receptive field enhancement mechanism and cascade architecture can have a great impact on the stable output of automatic segmentation results with high accuracy, which is critical for an algorithm. The final DSC, SEN, ASSD and HD95 values can be increased to 76.23 ± 6.45%, 79.14 ± 12.48%, 1.39 ± 5.44mm, 4.72 ± 3.04mm. In addition, the outcomes of multi-institution experiments demonstrate that our model is robust and generalizable and can achieve good performance through transfer learning.

**Conclusions:**

The proposed algorithm could accurately segment NPC in CT images from multi-institutional datasets and thereby may improve and facilitate clinical applications.

## 1 Introduction

Originating in the nasopharynx epithelium, NPC is a malignant tumor with the highest incidence among otolaryngological cancers in Southwest Asia, Southern China and Northern Africa (1, 2). Radiation therapy is the preferred treatment strategy for NPC because the poorly differentiated squamous cell carcinoma discovered upon pathological examination of patients with NPC is commonly radiosensitive (3). Delineating the tumor contour is the essential step in radiotherapy planning, which is the mainstay of NPC treatment. Based on the gross tumor volume (GTV) and organs at risk (OARs), the dose distribution of irradiation can be calculated by a radiation physicist. Therefore, accurate segmentation contributes to delivering the prescribed dose to the tumor volume while improving the sparing of OARs (4). However, in clinical practice, tumor segmentation is carried out manually by slices using multimodal or multiparametric imaging datasets, which is time-consuming (5, 6). Interobserver variability, especially in accuracy, is based on the expertise and experience of the radiation oncologist (7, 8) because on imaging, NPC often has a more complex tissue structure that has a similar intensity to its neighboring organs and a high variations in shape and size among cases (9). Accordingly, it is clinically desirable to develop a robust, accurate and automatic algorithm for target segmentation, which is helpful for reducing the labor intensity and interobserver variability.

Among many of the proposed autosegmentation approaches, atlas-based segmentation (10) has been widely used for the delineation of targets and/or OARs in head-and-neck radiotherapy (11–14), and it can obtain acceptable results without supervision. Currently, with the enormous success of deep learning in object detection (15), image classification (16), and segmentation (17), the applications of deep learning in medical imaging have received great attention. As the most popular algorithm for deep learning, convolutional neural networks (CNNs) have made significant progress in semantic segmentation with the advantage of an end-to-end framework for feature learning and model training. After the full convolutional network (FCN) was proposed by Long et al. (18), segmentation was achieved more efficiently in inference and learning for images with arbitrary sizes. UNet is the most successful FCNs utilized in medical image segmentation investigations and has been cited more than 29000 times since it was proposed in 2015 (17). Its success is largely attributed to the U-shaped architecture and skip connection in which the fusion of multiscale features and the recovery of fine-grained details can be realized effectively.

Deep learning with the CNN technique in tumor segmentation has recently made progress in the brain (19), rectum (20) and breast (21). For the segmentation of the GTV of NPC, a majority of CNN-based approaches have been applied in magnetic resonance imaging (MRI), which demonstrates superb resolution and soft tissue contrast and obtains satisfying results (22–24). However, radiotherapy plans are designed based on CT images, and MRI-based radiation therapy techniques have not been widely applied in clinical practice. Benefitting from the complementary information from both CT and MRI images, Ma et al. (25) proposed a multimodality segmentation framework based on CNN. While the performance of this kind of approach largely depends on the image registration accuracy, its difficulty will be further increased due to different body positions, scan times and imaging mechanisms. Therefore, the CT-based NPC segmentation technique is the core element that can actually solve the above clinical problems.

Although several methods have been explored for CT-based GTV segmentation (4, 26, 27), the results are barely satisfactory, and it remains the most challenging task primarily because of 1) CT images with lower contrast result in a lack of clear tumor boundaries; 2) tumors present a great variety of sizes and morphologies between different stages, especially for stages T3 and T4, which always have lymph node metastasis and other distant metastases, thus increasing the difficult of distinguishing the primary tumor. Therefore, the presented algorithms demonstrated good performance for early nasopharyngeal carcinoma, although the accuracy decreased sharply when advanced-stage tumors were included. Meanwhile, most of these methods were based on 2D segmentation models with a lack of complementary information between CT image layers. Notably, current research is based only on one specific institution dataset and does not consider differences between different data sources, which limits the universality of clinical applications.

Hence, we explored automatic delineation of GTV based on CT images and assessed its applicability for stages T1-T4. In our previous study (28), a modified version 3D U-Net model based on Res-block and SE-block to delineate the GTV for NPC was developed; however, the accuracy still needs to be improved. With the aim of promoting network performance, we propose a cascaded multiscale local enhancement CNN structure, which can realize NPC segmentation from global to local scales by concentrating on the GTV region, and multiscale features in CT images can be captured simultaneously. Comprehensive experiments on diverse multi-institutional planning CT datasets were performed to demonstrate the effectiveness of our algorithm. Both the qualitative and quantitative evaluation results show that our approach can achieve good segmentation performance and outperforms other state-of-the-art segmentation methods.

## 2 Materials And Methods

### 2.A. Datasets

We collected retrospective data on 257 patients who were diagnosed with NPC with stage T1-T4 and underwent radiotherapy in our institution from 2016 to 2020. Data were derived from radiotherapy treatment planning, including plain CT (pCT) and contrast-enhanced CT (CE-CT) images, with the scanned region covering the overall head, neck and partial chest obtained by simulation CT. All images were axially reconstructed with a matrix size of 512 × 512 pixels, a resolution of 0.748~0.976 mm and a slice thickness of 3.0 mm. The radiotherapy contours were jointly delineated slice by slice by two radiation oncologists on the pCT image by fusion of CE-CT and MR images according to the consensus as the ground truth. The director with 20 years’ experience of the radiation oncology department was consulted in cases of disagreement.

Meanwhile, multi-institutional datasets were also employed in the experiments to evaluate the performance of the proposed algorithm. These datasets from institution B are composed of 40 NPC patient pCT images, and the MICCAI 2019 StructSeg challenge (GTV segmentation task) is composed of 50 NPC patient pCT images. **Table 1** shows the details of the multi-institutional datasets.

**Table 1:** Details of the multi-institutional datasets.

Dataset	A	B	C
**Source**	Our department	Institution B	MICCAI 2019
**Images**	pCT + CE-CT	pCT	pCT
**Number**	257	40	50
**Stage**	T1-T4 (30:93:87:47)	NA	NA
**Axially size**	512 × 512	512 × 512	512 × 512
**Slice thickness**	3 mm	3 mm	3 mm

### 2.B. Overview of the Method

**Figure 1:**
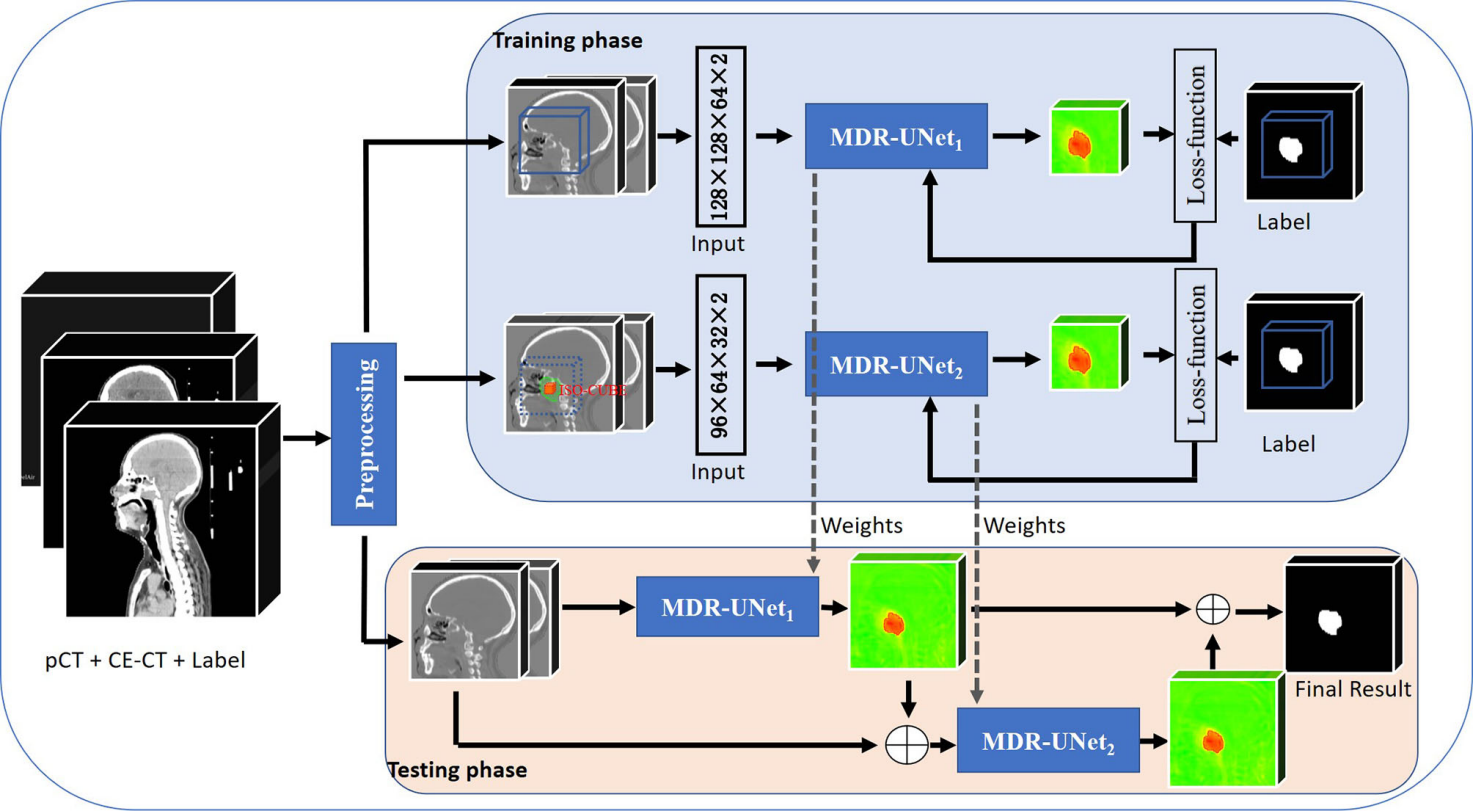
Overview of the workflow of the proposed method.

In this paper, inspired by cascaded method (29), we propose a two-stage cascaded multiscale local enhancement network structure to achieve the goal of building a precise GTV segmentation method. In the training phase, two networks were trained simultaneously: one was for globally coarse segmentation predictions and named MDR-UNet_1_, and the other was for locally fine segmentation predictions concentrated on the GTV and named MDR-Uet_2_. The testing phase could be divided into three steps: 1) Obtain the globally coarse segmentation result by MDR-UNet_1_; 2) Identify the central location of the ROI from coarse results and acquire precise segmentation for the cropped target region by MDR-UNet_2_; and 3) Assemble the two-stage results to output as the final prediction. The overall framework is presented in **Figure 1**.

The above two-stage independent cascade network structure with central localization was designed to achieve ‘coarse-to-fine’ segmentation for the GTV region. Moreover, there are two other key points that should be noted in this method: 1) Crop the ROI using a body mask, which is obtained by morphology and geometry; and 2) Use multiscale dilated convolution blocks in the skip connections between the encoder and decoder to enhance the receptive field to improve the segmentation performance. These points will be described specifically in the following.

### 2.C. Preprocessing

In this study, a 3D CNN was introduced in our automated delineation GTV network structure. However, calculation efficiency was an issue because of the massive occupation of graphic processing unit (GPU) memory caused by 3D data. Reducing neural network channels, layers, and batch size are always selected as the solution; however, such changes will have a great negative impact on the training results. Hence, we preprocessed the data to minimize the data cropped from the original images and simultaneously guarantee that the target was completely included. For the preprocessing procedures, we first removed beds and obtained the body mask by utilizing Hough transform line detection, threshold and morphological methods, which are binary images with targets shown in pixels (value=1) and ground shown in pixels (value=0). Second, the body mask was integrated along the vertical direction to find the minimum point, and the corresponding abscissa was the neck position. Then, the volume above the neck was clipped as the ROI. Finally, for normalization, 1) the target voxel spacing was normalized to 0.952×0.952×3 mm for all of the data with third-order spline interpolation; and 2) the intensities were normalized by subtraction of the global mean and division by the global standard deviation, which are obtained by computing the foreground voxels based on body mask cropping in the dataset. After preprocessing, the image overall mean size with standard deviation decreased from 512×512× (130 ± 11.7) to (190 ± 32.8) × (222 ± 17.4) × (74 ± 4.8).

### 2.D. Proposed Two-Stage Cascade Architecture

#### 2.D.1. Stage 1: Initial Coarse Segmentation and Central Localization

Although the image size was greatly reduced to half of the original after preprocessing, it was still too large to put all the images into the network for 3D CNN training. Therefore, the preprocessed images were randomly cropped by a sliding window before being put into the network during the training stage to satisfy the calculation requirement. However, although the cropped image might contain massive background information, a few ROIs result from random cutting. As a result, only the globally coarse segmentation region of the GTV has been extracted at this stage. Based on the preliminary segmentation, the centroid of the GTV can be determined and used as preparation for the next locally precise segmentation. As a trade-off between model performance and memory consumption, the size of the window was set to 128×128×64.

#### 2.D.2. Stage 2: Central ROI Fine-Segmentation

The aim of this stage was to further narrow the target region and perform precise segmentation around the center of the tumor. To achieve this goal, the first step was to realize the central localization of the tumor. For the training data, the center of the GTV can be identified from the ground truth, while for the test data, the location can only be determined from the segmentation predicted in stage 1. Considering the error between them, the position differences for the training data were compared, and the results demonstrated that the centroid of the GTV between the ground truth and stage 1 segmentation results varied (1.46 ± 1.34, 1.84 ± 1.69, 1.14 ± 1.09) pixels. Hence, by considering the error, we allowed the central location to vary with the range in ( ± 3, ± 3, ± 3) pixels of the center determined from the ground truth or stage 1 segmentation result. In addition to the central localization, the cropped cube size of the GTV had to be determined. The cube size of the GTV is known to correlate with the stage of the tumor; therefore, we calculated the size of the GTV for all of the data and found that the overall mean with standard deviation was 64 ± 8.5, 43 ± 12.1, and 15 ± 5.7 pixels. Thus, the cropped cube size of the ROI was set to 96×64×32. The process not only made it possible to further reduce the size of the input image, which contains much more ROI area and less background information, but also optimized the network model and facilitated its convergence in training.

#### 2.D.3. Network Architecture

**Figure 2:**
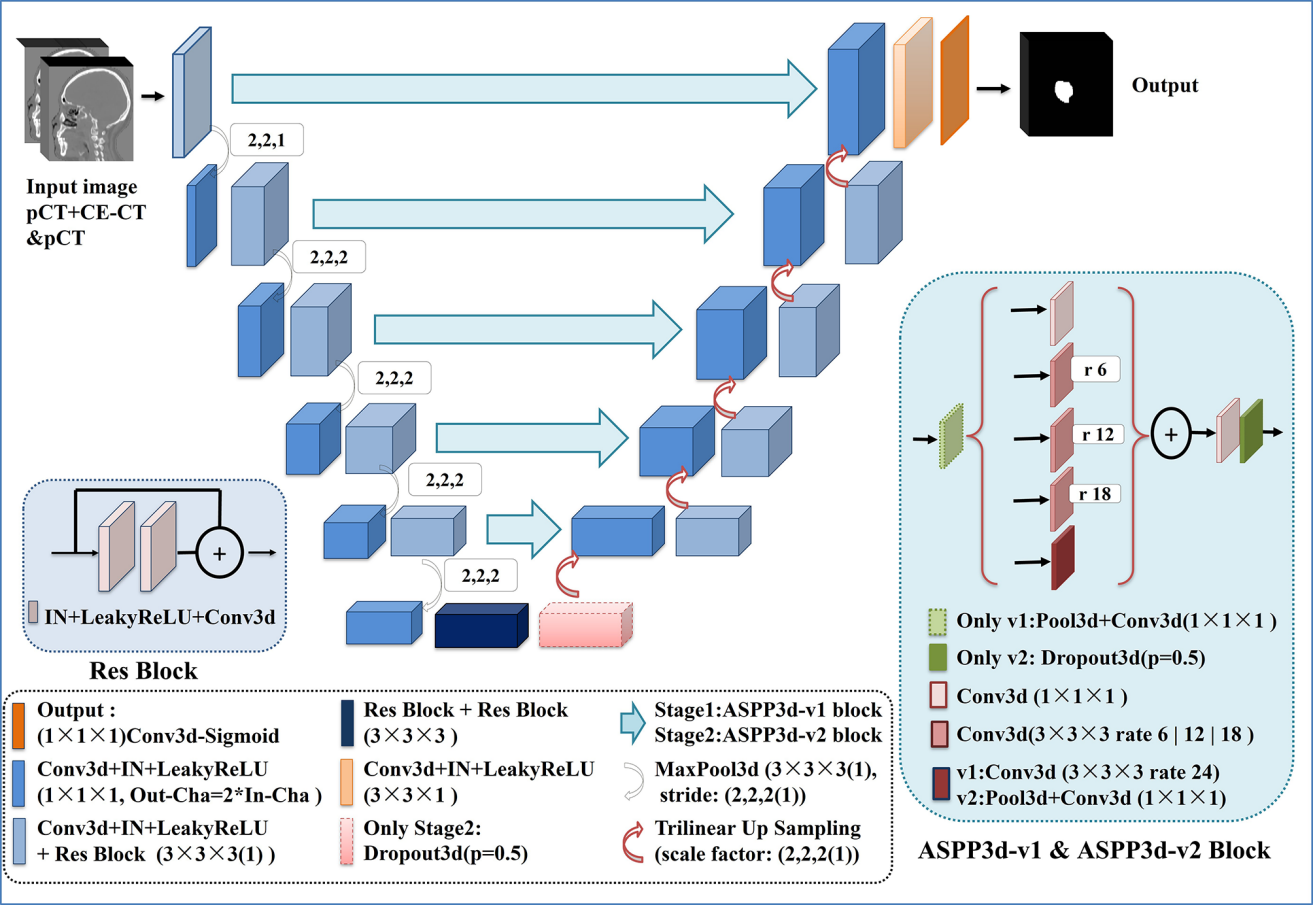
Network architecture of the proposed CNN model.

Our network architecture is shown in **Figure 2**. We used UNet (17) and its 3D counterpart VNet (30)-like architecture as the backbone due to their excellent performance in medical image segmentation. UNet consists of three components: 1) downsampling for feature extraction, 2) upsampling for resolution restoration and 3) skip-connection for feature fusion, which can achieve the multiscale feature extraction of large medical images. Res-block (31) was introduced in the procedure of downsampling and upsampling because it has excellent performance in deep networks for solving gradient dispersion and precision reduction. In the skip connection, we employed the modified 3D ASPP (32, 33) block because it can use multiscale dilated convolution to enlarge the field of view. The proposed network contains 11 Res-blocks and 5 ASPP-blocks in total. The network adopts the common configuration of blocks per resolution step in both the encoder and decoder, with each block consisting of a Conv-block and Res-block. The proposed network can only be trained with a small batch size for a large patch size. In the case of a small batch size, we utilized instance normalization (34) to speed up or stabilize the training because of the poor performance shown by batch normalization (35). Furthermore, the leaky ReLU (36) was used as the activation function in the hidden layers. Following the final segmentation map, sigmoid activation function outputs are obtained. As a compromise between model performance and memory consumption, the initial number of feature maps was set to 32 and doubled or halved in each downsampling or upsampling procedure. To limit the parameter size of the final model, the number of feature maps was additionally capped at 512 and 1024 for stage 1 and stage 2, respectively.

For the best performance, the two-stage network architectures were slightly different to allow for adaptations to different data characteristics. In stage 1, due to the large input image, we introduced the ASPP block (32) with a larger dilation rate in the skip connection to obtain multiscale feature information of different fields of view. For stage 2, further reducing the size and range of the input image would contribute to the appearance of overfitting during the network training. Hence, to obtain a better training effect, the ASPP_v3+ block (33), which has a small dilation rate and includes a dropout layer, was employed to replace the ASPP block in the skip connection for this stage. In addition, the dropout layer was also added at the end of the encoder to prevent overfitting.

### 2.E. Postprocessing

In the test phase, to obtain the initial coarse segmentation result, the test samples were input into the stage 1 network by grid sliding with an input size of 128×128×64 and a stride of 64 × 64 × 32. The stage 2 input ROI could be extracted from the original image by taking the centroid of the GTV determined from the stage 1 segmentation result as the center of a cube. For both stage networks, the segmentation results were acquired after activation with a sigmoid function. Then, the segmentation results obtained in stage 1 and stage 2 were assembled by S =*ω*S_1_ + (1 – *ω*)*S*_2_, ω was set to 0.5, and the final segmentation score map was output, where the threshold value was set to 0.6 and values lower than the threshold were regarded as background. Finally, the predicted ROI was reconstructed and returned to the corresponding position of the original CT images.

## 3 Experiments And Results

### 3.A. Experimental Scheme

#### 3.A.1 Implementation Details

Fivefold cross-validation was adopted to evaluate the network performance among our 257-patient dataset, which meant that the dataset was randomly divided into 5 subsamples. Specifically, in each round, one of the subsamples (20%) was used as the test set and the remaining four folds (80%) were used as training set. We repeated this procedure five times until all the five folds have been used as the test set. Varieties of data augmentation techniques were introduced into the training dataset, and they consisted of random scaling (scaling factors: 0.7~1.3), random elastic transformation (scaling factor: 34; elasticity coefficient: 10), random rotation (angle: -10~10), random noise (Gaussian noise: 0~0.1 or uniform noise: -0.1~0.1), and random flipping.

In our 257 dataset, for the two networks, the ‘kaiming normal’ (37) strategy was employed for weight parameter initialization, the stochastic gradient descent (SGD) optimizer was used for training with momentum of 0.99, the learning rate (LR) abided by the ‘poly’ policy (32) decaying with LR = lr_ini_* (1 –*epoch*/*epoch*_max_)^0.9^, initial learning rate (lr_ini_) was set to 0.01, and the loss function was the sum of cross-entropy and dice loss. Based on previous experiments and experience, as a compromise between runtime and reward, each fold of both networks was set to 120 epochs. The batch size was set to 1, and the network parameters were updated every 2 batch sizes with the gradient accumulation method.

The experiments were implemented on a workstation powered by a NVIDIA GeForce RTX 2080Ti with 11 GB GPU memory. The code was implemented with Pytorch 1.4.0 in Ubuntu 18.04.3 LTS.

#### 3.A.2 Evaluation Criteria

Three volumetric overlap metrics, the Dice similarity coefficient (DSC), positive predictive value (PPV), and sensitivity (SEN), and two distance metrics, the average symmetric surface distance (ASSD) and 95% Hausdorff distance (HD95), were utilized as qualitative evaluation criteria to estimate the performance of our automated segmentation algorithm. These metrics are defined as follows.

a) Volumetric overlap metrics:


(1) $$DSC=\frac{2|G\cap A|}{\left|G|+|A\right|}$$

(2) $$PPR=\frac{\left|G\cap A\right|}{\left|A\right|}$$

(3) $$SEN=\frac{\left|G\cap A\right|}{\left|G\right|}$$

b) Distance metrics:


(4) $$ASSD=\frac{1}{2}\left\{\underset{g\in G_{s}}{\text{mean}}\underset{a\in A_{s}}{\min}\;d(a,g)+\underset{a\in A_{s}}{\text{mean}}\underset{g\in G_{s}}{\min}\;d(a,g)\right\}$$

(5) $$HD_{95}(G_{s},A_{s})=\max(d_{95}(G_{s},A_{s}),d_{95}(A_{s},G_{s}))$$

Where


(6) $$d_{95}(G_{s},A_{s})=K_{g\in G_{s}}^{95}(\underset{a\in A_{s}}{\min}||a-g||)$$

For the volumetric overlap metrics, G and A represent the voxel sets of the ground truth and automatic delineation, respectively. DSC, PPV and SEN, which are a series of ratios, are used to describe the corresponding spatial overlap between the ground truth and the automated delineation, and a higher value indicates better performance.

For distance metrics, where *G_s_* and *A_s_* are the corresponding surface voxel sets of *G* and *A*, *d*(*a,g*) and ||a – *g*|| are the Euclidean distance of the voxel between *a* and *g*, $d_{G_{s}A_{s}}$ describes the point *x* ∈ *X_s_* that is farthest from any point of *Y_s_* and calculates the distance from *x* to its nearest neighbor in *Y_s_*. ASSD and HD95 describe the mean surface distance between the ground truth and automated delineation, and a lower value shows a higher delineation accuracy.

In addition, IBM SPSS (version 23; New York, NY) was used for statistical analysis. The mean DSC, PPR, SEN, ASSD and HD95 values for the GTV segmentation were evaluated the dispersion with standard deviation (SD) and analyzed with paired t-tests between different models. All values are presented as mean ± SD. Two-tailed p-values <0.05 were considered statistically significant.

#### 3.A.3 Comparison of Model Performance

Automated segmentation models of the GTV based on the proposed cascaded multiscale local enhancement CNN structure were achieved in this study. First, several successful network architectures and our proposed 3D Res-UNet without ASPP block (33) and cascade architecture were applied to data for 257 NPC patient cases from our institution for GTV segmentation to compare the performance. Two modalities (pCT and CE-CT) were used to train the network models, because the advantage of CE-CT in terms of contrast visibility between tumor and normal tissues can help improve the performance of network segmentation (28). The other successful network architectures were as follows: 3D CNN (22), which is based on the 3D CNN architecture of VoxResNet and used to segment the complicated NPC GTV based on MRI showing outstanding performance, Attention-UNet (38), which adds AGs to filter the features propagated through the skip connections of UNet; and UNet++ (39), which improves UNet performance by alleviating the unknown network depth, redesigning the skip connections, and devising a pruning scheme to the architecture. For comparison, the Attention-UNet and UNet++ were changed from 2D to 3D network architectures. Second, based on the 3D Res-UNet backbone model, the important roles of the multiscale dilated convolution blocks and cascade architecture, namely, the five ASPP blocks and the two-stage cascade method shown in **Figure 2**, were further explored experimentally in the proposed algorithm. Moreover, the feature maps and the boundary probability maps were output further to confirm the ASPP block mechanism on segmentation performance.

Finally, additional datasets of 40 NPC patients from institution B and 50 NPC patients from the MICCAI 2019 StructSeg challenge were also adopted to validate the effectiveness of the model further and evaluate the generalizability of the model application. The model was applied to two additional datasets for further algorithm verification experiments in MDR-UNet without the cascade architecture to reduce time consumption. Because of the lack of CE-CT data in the institution B and C datasets, only pCT data were used for training. It is important to note that transfer learning was used to fine-tune the network based on the model trained to reduce estimation errors between different datasets. Specifically, the model was first trained on the A dataset, and its performance was evaluated on the B and C datasets. Then, based on the model pre-trained on the A dataset, transfer learning was employed for training datasets B, C and B+C. Moreover, the original and target domains were switched in the experiment to verify further the generalization ability of the network in transfer learning. Datasets B, C, and B+C were utilized for training the network to verify the network performance and tested on the A dataset. The network was then fine-tuned with dataset A using the transfer learning method. It should be noted that during transfer learning stage, datasets A, B and C were randomly split into 70% and 30% respectively for fine-tuning and testing. The network was trained for 60 epochs, and the initial learning rate lr_ini_ was adjusted to 0.001 during the fine-tuning phase.

### 3.B. Results and Analysis

#### 3.B.1. Comparison Between Different Network Architectures

**Figure 3:**
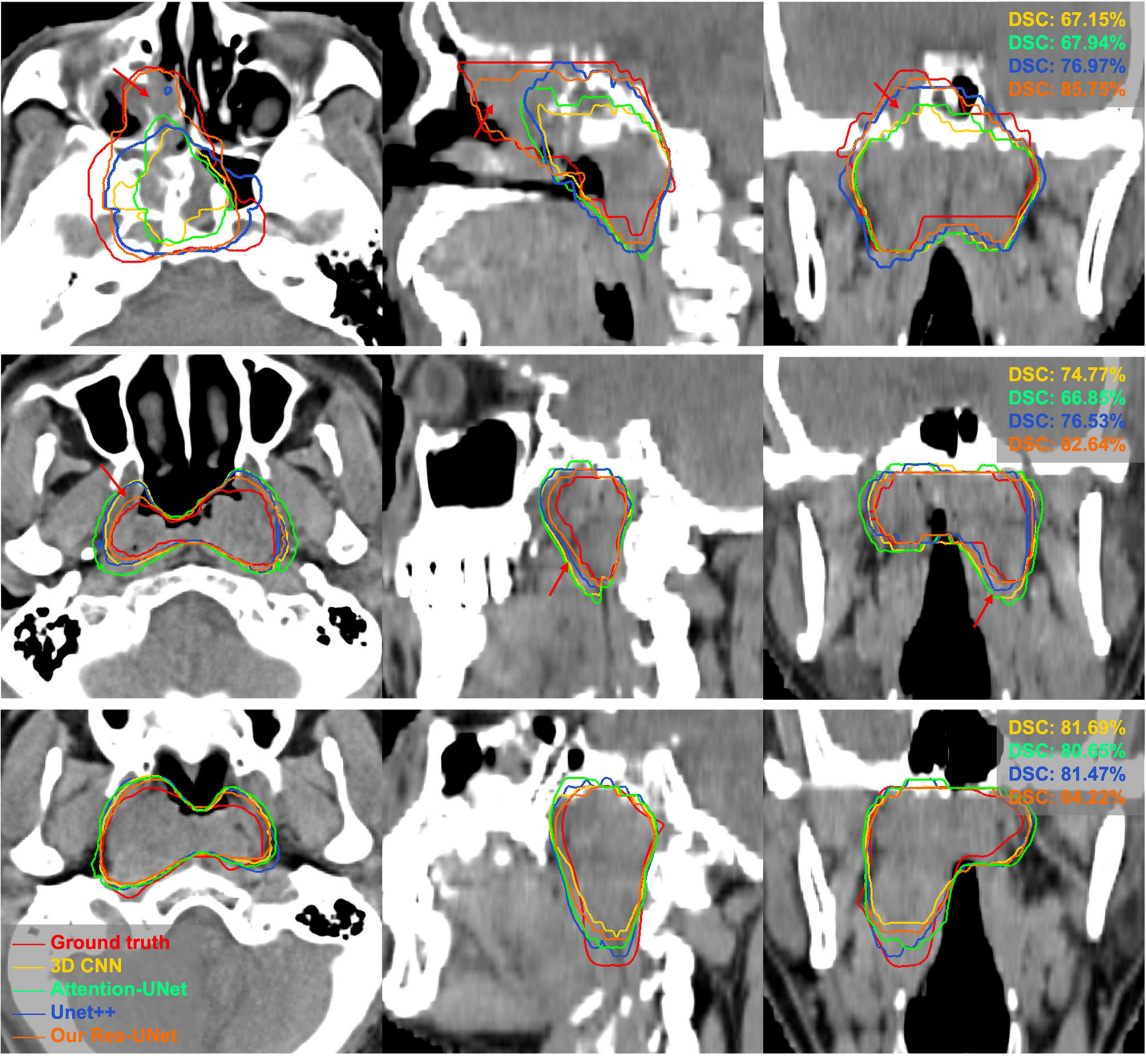
Visual comparison of different networks for GTV segmentation. The red arrows denote false positives and poorly segmented areas. Note that these results derived from the models trained on pCT and CE-CT data are shown in pCT.

The quantitative comparison results of the fivefold cross-validation of the different network models trained by pCT and CE-CT are summarized in **Table 2**. As shown in the table, Res-UNet performed better in mean DSC, PPR, ASSD and HD95 with standard deviations of 74.49 ± 7.81%, 79.97 ± 13.9%, 1.49 ± 0.65mm, 5.06 ± 3.30mm, respectively, compared to other networks, benefiting from the dominance of the RES-block in solving gradient dispersion and precision reduction, which verifies the effectiveness of the proposed backbone. However, the mean SEN index of Res-UNet was only 73.9%, which is a poor result due to the lack of relevant attention mechanism, indicating that the network is under-segmented. **Figure 3** shows the visual segmentation results for the comparison of automatic ground truth delineation by the public networks, the method used for this research and human experts. It is obvious that the estimation of the proposed Res-UNet produces fewer false positive predictions and presents more robust segmentation results, particularly in the coronal and sagittal views, compared to the 3D CNN, 3D Attention-UNet and 3D UNet++. To summarize, the experimental results demonstrate that although the Res-UNet backbone network might not have the best performance and statistical significance in all indicators. Nevertheless, the key indicator DSC was significantly improved (P<0.05) and had better performance than other successful architectures when dealing with anisotropic 3D resolution.

**Table 2:** Quantitative comparison of different backbone models for GTV segmentation performance, including mean DSC, PPR, SEN, ASSD and HD95 with standard deviation.

Method/P -value		DSC (%)	PPR (%)	SEN (%)	ASSD (mm)	HD95 (mm)
① 3D CNN (22)		73.67 ± 7.88	76.74 ± 14.71	75.27 ± 14.31	1.84 ± 3.91	6.32 ± 13.77
② 3D Attention-UNet (38)		73.54 ± 7.16	75.95 ± 14.59	**76.04 ± 14.48**	1.80 ± 1.64	6.74 ± 11.91
③ 3D UNet++ (39)		73.87 ± 7.07	77.73 ± 14.40	74.82 ± 14.26	1.53 ± 0.60	5.17 ± 3.04
④ Proposed 3D Res-UNet		**74.49 ± 7.81**	**79.97 ± 13.90**	73.90 ± 14.58	**1.49 ± 0.65**	**5.06 ± 3.30**
P-value	④ *vs.* ①	0.026*	<0.001*	0.041*	0.179	0.190
	④ *vs.* ②	0.007*	<0.001*	0.001*	0.005*	0.043*
	④ *vs.* ③	0.027*	<0.001*	0.107	0.213	0.520

Asterisks (∗) indicate that the difference between the proposed 3D Res-UNet method and the competing method is statistically significant (p < 0.05) using a paired t-test. The best result is highlighted in bold.

#### 3.B.2. Evaluation of the Network Mechanism

**Figure 4:**
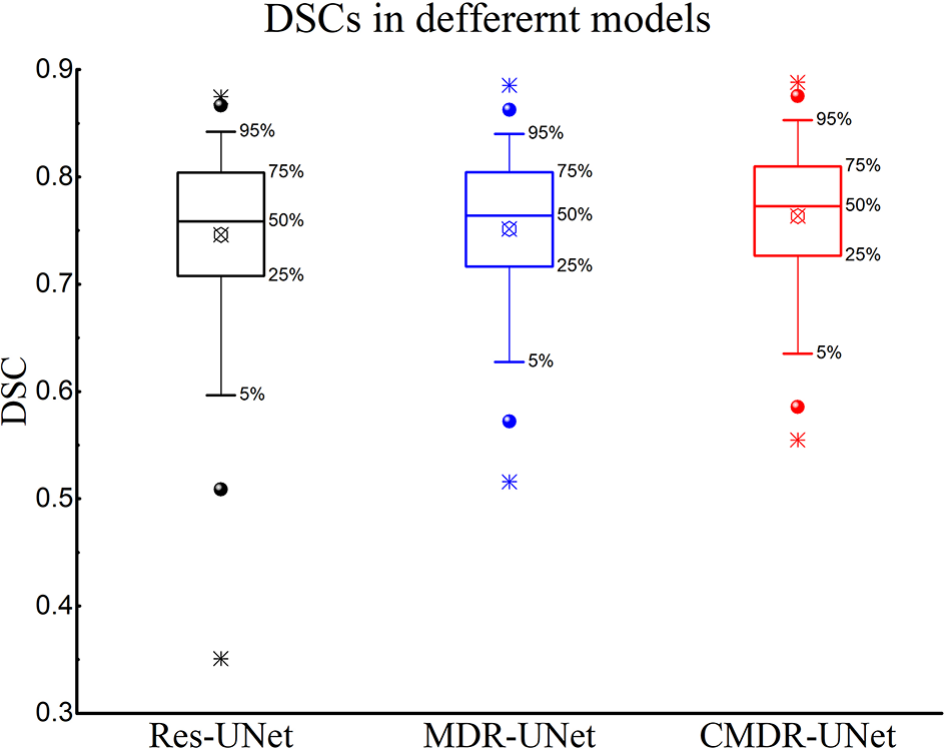
Boxplot showing the DSCs of different models. The symbols 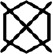 represents the means, 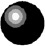 represents the 1% and 99%, 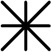 represents the min and max.

When the 3D Res-UNet backbone was combined with the ASPP blocks and cascade architecture, the experimental results trained by pCT and CE-CT illustrated significant improvements in most of the evaluation indicators under the effect of local enhancement mechanisms, as shown in **Table 3**. The mean values of DSC, SEN, ASSD and HD95 increased to 76.23 ± 6.45%, 79.14 ± 12.48%, 1.39 ± 5.44mm and 4.72 ± 3.04mm. It is found that the mean PPR decreased from 79.97 ± 13.90% to 77.34 ± 14.04% (P<0.001), while the mean SEN increased from 73.90 ± 14.58% to 79.14 ± 12.48% (p<0.001). Although the network model changed from under-segmentation to over-segmentation, the model was able to keep the segmentation results in a relatively optimal state. For DSC, which is the key indicator of segmentation performance, a boxplot is shown in **Figure 4**. The differences can be found in the following three statistical intervals, i.e., the 5%-95%, 25%-75% and 50%, and the results show that the model with the ASPP block and cascade architecture, named CMDR-UNet, has a higher average value along with a smaller variance (P<0.001).

**Table 3:** Comparison of the effects of adding the ASPP blocks and the proposed cascade architecture.

Method/P -value		DSC (%)	PPR (%)	SEN (%)	ASSD (mm)	HD95 (mm)
① Res-UNet		74.49 ± 7.81	**79.97 ± 13.90**	73.90 ± 14.58	1.49 ± 0.65	5.06 ± 3.30
② MDR-UNet		75.16 ± 6.76	78.73 ± 13.50	75.81 ± 13.65	1.68 ± 3.44	5.82 ± 13.57
③ CMDR-UNet		7**6.23 ± 6.45**	77.34 ± 14.05	**79.14 ± 12.48**	**1.39 ± 5.44**	**4.72 ± 3.04**
P-value	② *vs.* ①	0.065	0.002*	<0.001*	0.409	0.418
	③ *vs.* ②	<0.001*	<0.001*	<0.001*	0.233	0.244
	③ *vs.* ①	<0.001*	<0.001*	<0.001*	<0.001*	0.002*

Two-tailed p-values <0.05 were considered statistically significant between the proposed different models using paired t-tests. The best result is highlighted in bold.

∗p < 0.05 was considered significant. The values were represented as mean ± standard deviation. MDR-UNet: Adding the multiscale dilate CNN of the ASPP module. CMDR-UNet: Adding our proposed cascade architecture.

**Figure 5:**
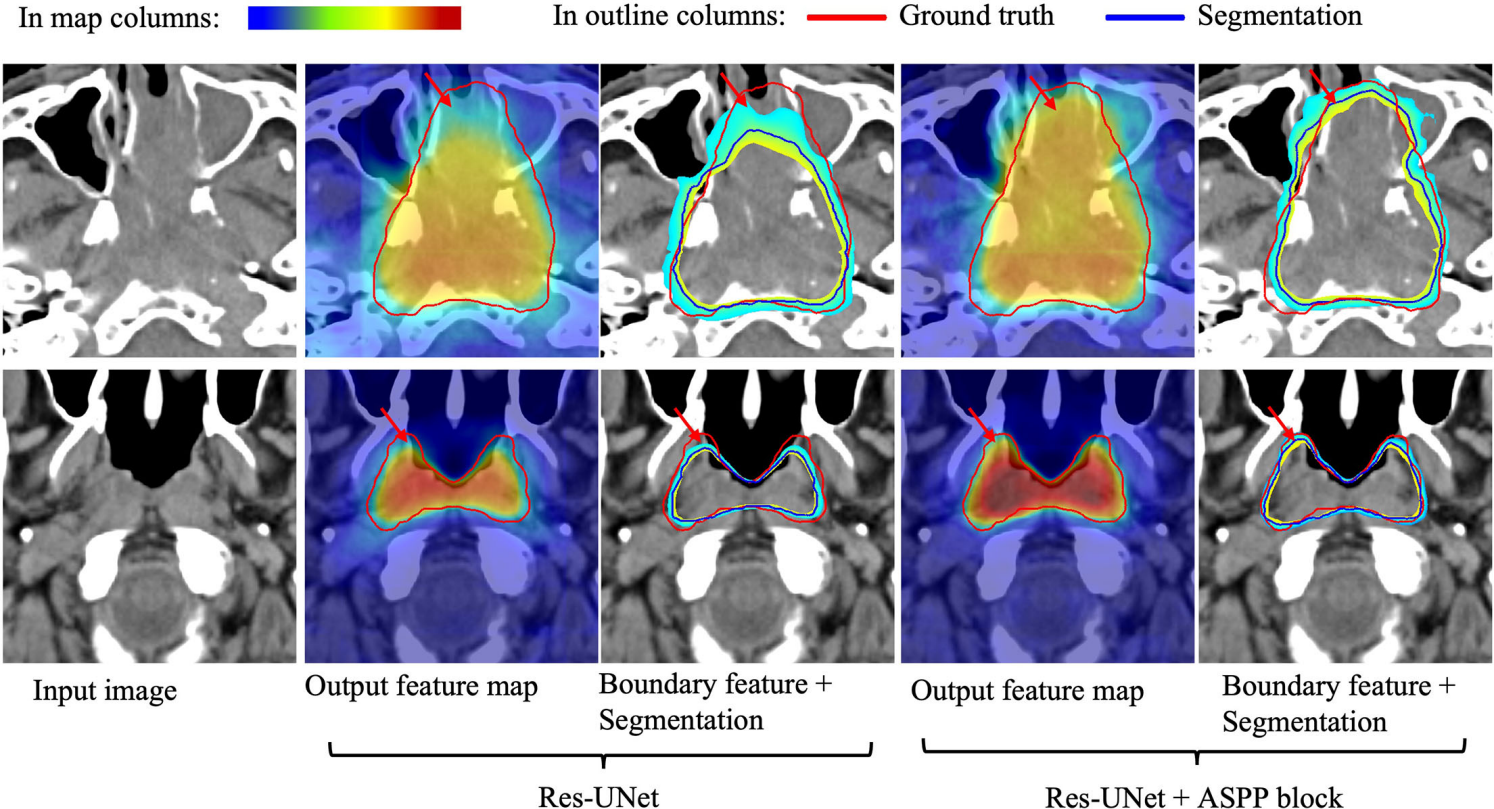
Visual comparison of the boundary feature maps obtained by including the Res-UNet backbone and adding the ASPP block. Map columns: includes output feature maps and boundary feature maps, where warmer colors represent higher attention. Outline columns: red and blue denote the ground truth and automatic segmentation results, respectively. The red arrows denote boundary areas noticed by the ASPP block, which has better boundary segmentation performance. Note that the two rows are from different patients and shown in pCT.

**Figure 6:**
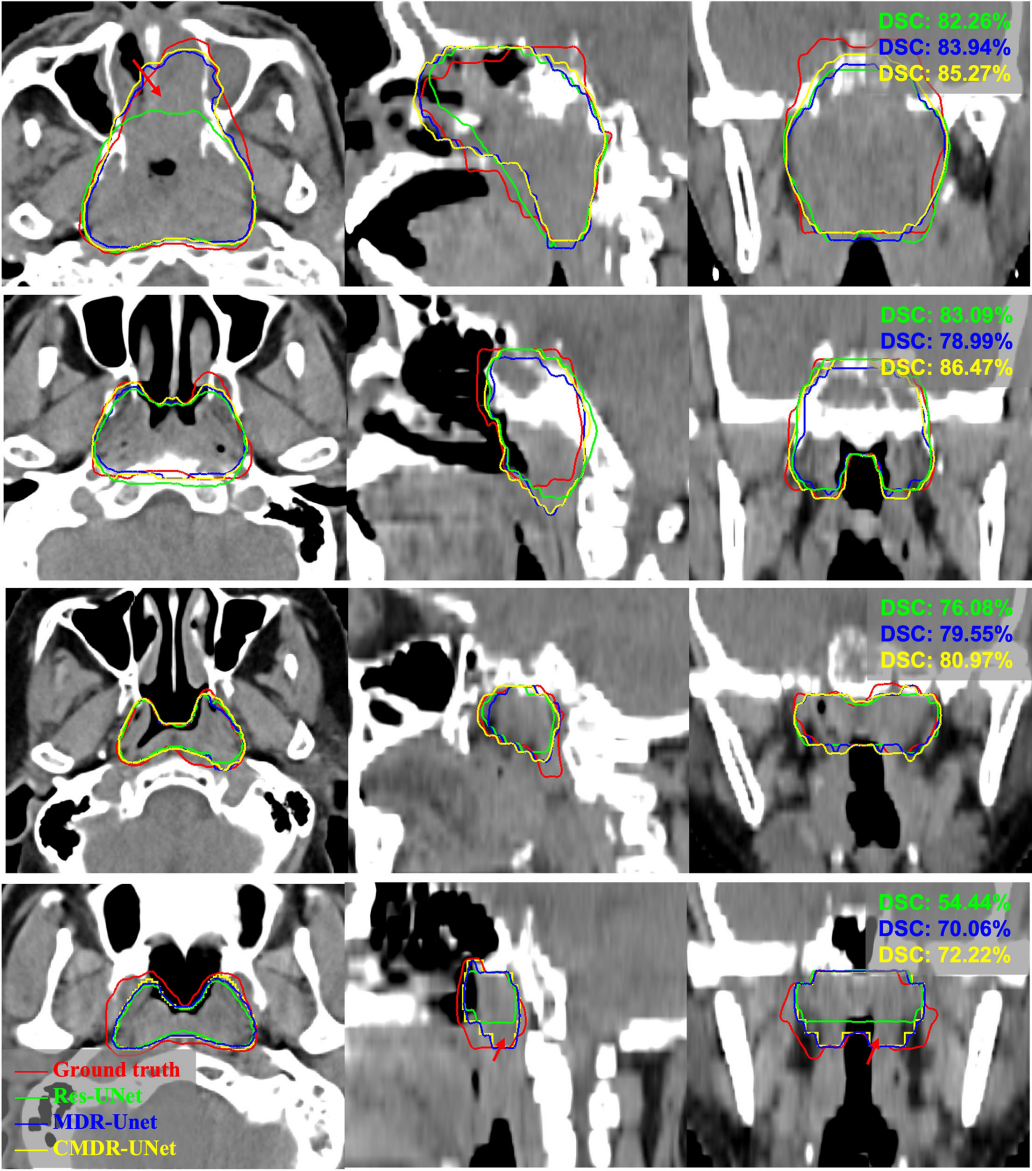
Example pCT images show the level of consistency for GTVnx between the automatic delineation with our method and the ground truth. Red lines denote the human expert-delineated ground truth, and the other lines denote the contours of the automatic delineation.

To further validate our network mechanism, we extracted two representative examples for boundary segmentation feature maps as shown in **Figure 5**. Because of the effect of the ASPP module, the weight distribution of the feature map is relatively uniform and the tumor boundary is sharp, which are indicated by the red arrows, when compared with the 3D Res-UNet backbone. **Figure 6** shows the visual comparison results of the three different modules for four instances of NPC segmentation along with the DSC quantification results. We found that the ASPP block is more sensitive to the boundaries of the GTV than the Res-UNet backbone, as shown by the red arrows indicating positions, while the cascade architecture further improves the stability of the network. In summary, the multiscale dilated convolution block and proposed cascade architecture can make a large difference in stably outputting automatic segmentation results with high accuracy, which is critical for an algorithm.

#### 3.B.3. Multi-Institution Experiments

The quantitative segmentation results of the models pretrained, fine-tuned and validated on the multi-institution datasets using mean DSC and ASSD with standard deviations were listed in **Table 4**. For the A(pCT) pretrained model, the mean results of the DSC and ASSD for the data with five-fold cross-validation were 74.63% and 1.58 mm, respectively. The DSC value was inferior to the 75.16% obtained for the DSC trained with pCT and CE-CT, as shown in **Table 3**. In these five models pretrained on dataset A, the model with the best performance was applied to the datasets B, C and B+C, the mean DSC and ASSD results were 68.21% and 2.09 mm, 61.64% and 2.13 mm and 64.26% and 2.12mm, respectively. Clearly, these results were not satisfactory. Then this pretrained model was fine-tuned with 70% of datasets B, C and B+C and the remaining 30% dataset was utilized for test, respectively. The mean DSC and ASSD results reached to 74.49%, 1.87 mm and 73.95%, 1.89mm for institution B, and 69.64%, 1.42 mm and 67.43%, 1.60mm for institution C, respectively. Compared with the model fine-tuned on the single dataset B or C, the evaluation metrics of model fine-tuned on the mixed dataset B+C showed slightly lower performance, which was probably caused by the pCT scanned from different devices and particularly the ground truth delineated by different doctors. Moreover, the problem of sample shortage leaded to the lack of stability in statistical differences. In summary, these results demonstrate that transfer learning can achieve good performance on external datasets.

**Table 4:** The segmentation results of the models pretrained, fine-tuned and validated on the dataset A, B, C and B+C.

Training datasets	Test datasets	DSC (%)	ASSD (mm)
A (pCT)	A	**74.63 ± 7.05**	**1.58 ± 0.94**
	B	68.21 ± 5.51	2.09 ± 0.74
	C	61.64 ± 13.55	2.13 ± 1.02
	B+C	64.26 ± 11.54	2.12 ± 0.93
A Pretrained + B (70%) fine-tuning	① B (30%)	**74.49 ± 6.86**	**1.87 ± 0.67**
A Pretrained + C (70%) fine-tuning	① C (30%)	**69.64 ± 10.02**	**1.42 ± 0.36**
A Pretrained + (B+C) (70%) fine-tuning	② B (30%)	73.95 ± 8.66	1.89 ± 0.78
	② C (30%)	67.43 ± 12.35	1.60 ± 0.49
P-value	② B *vs.* ① B	0.810	0.893
	② C *vs.* ① C	0.180	0.041*

Two-tailed p-values <0.05 were considered statistically significant. The best result is highlighted in bold.

The values were represented as mean ± standard deviation. ∗p < 0.05 was considered significant. A: Our institution; B: institution B; and C: MICCAI2019.

To further test the robustness and transferability of the model application, the original and target domains were switched for the following experiments. The segmentation results of the models pretrained, fine-tuned and validated on the dataset B, C, B+C and A were listed in the **Supplementary Table 1**. The pretrained network model achieved good performance on the B and C datasets with the mean DSC and ASSD of 72.84%, 1.89 mm and 65.77%, 2.56mm, respectively. It shows that the model also has good robustness on other datasets. However, direct application of the trained models based on B and C datasets to the A dataset shows poor performance with mean DSC and ASSD as low as 61.23%, 2.80mm and 58.88%, 2.72mm, respectively. Meanwhile, dataset A (70%) was used to fine-tune the models pretrained on the B, C and B+C datasets, respectively. The mean DSC and ASSD results reached 74.81%, 1.36mm, 74.27%, 1.40mm and 74.46%, 1.61mm, respectively. It is easy to notice that there is no improvement in the above results compared to the dataset directly training results of 74.46%, 1.45mm (P>0.05). These results suggest that models trained on small datasets have poor generalization ability and transfer learning being introduced to large datasets may not improve performance.

## 4 Discussion And Conclusion

Blurred tumor boundaries and large shape variations have always been huge challenges for GTV segmentation of NPC in lower-contrast CT images. In this work, a 3D automatic segmentation algorithm has been proposed to solve these issues, in which the concept of multiscale local enhancement is employed with the foundation of the 3D Res-UNet backbone model. First, for small target areas, such as GTV, we introduced the multiscale ASPP-block in skip-connection to guide the network to focus more on the target area, especially for the boundary during learning, to promote the segmentation performance. Second, benefiting from the excellent ability to capture both global and local feature information simultaneously, a cascade architecture was adopted in training for outputting robust segmentation results. Moreover, we adopted an automatic preprocessing method to reduce the image background to solve the problem of heavy computation and memory consumption in loading the 3D network model data.

In multi-institution experiments, as shown in **Tables 3**, **4**, CE-CT is helpful for improving segmentation performance. Due to the differences in data sources and manual delineation for different institutions, as demonstrated in the literature (22), DSCs can vary from 71% to 80% compared to the manual contours of eight radiation oncologists with ground truth contours. In our experiments, it was found that direct application of the model trained with the internal dataset to the external dataset yielded suboptimal results. However, the performance of the models can be significantly improved by transfer learning, which only requires fine-tuning the network with a smaller dataset to output comparable results obtained from a larger dataset. Although transfer learning may not be used to improve the performance of applying a model trained on a small dataset to the results of a large dataset, it can output comparable results with higher training speed. In addition, the models were applied to the small datasets B and MICCAI, where the DSC could reach 72.84% and 65.77%, respectively. Another study based on MICCAI data showed that the DSC of the ensemble multi-scale model was only 65.66% (40), which is comparable to the result (65.77%) but lower than the proposed method with transfer learning (69.64%). It shows that the model is robust to small datasets. In general, although a trained model cannot not be valid for all dataset estimations, the proposed model can achieve good performance through transfer learning.

Indeed, limitations have been observed within the current model. In the multi-institution experiment, since the labels are delineated by different radiation oncologists, the dataset cannot be evaluated uniformly when multiple institutions are integrated into the network model for supervised training. In future work, the segmentation performance will be further improved in the following three aspects: 1) To overcome the differences in tumor delineations by various doctors, the accuracy and consistency of delineation will be further advanced. 2) Considering the significant differences between numerous data sources, a semi-supervised method will be introduced in training to enhance the robustness of the network. 3) Multimodal data will be utilized to assist CT image segmentation.

Extensive experiments on our CT dataset show that our proposed CMDR-UNet method based on the modified 3D Res-UNet backbone outperforms other state-of-the-art methods and achieves the best results for the quantitative indicators DSC, PPR, ASSD and HD95. In multi-institution experiments, due to the differences in data sources and manual delineations for different institutions, the segmentation results of other datasets acquired from a single institution trained model is unsatisfactory, although this issue can be resolved by transfer learning. This finding is desirable because it partially reflects the universality of our proposed algorithm for clinical application.

## Data Availability Statement

The data analyzed in this study is subject to the following licenses/restrictions: The datasets for this article are not publicly available because the data were collected by multiple centers and publicly accessible data is not approved. Requests to access these datasets should be directed to Zhenhui Dai, dzh_dzh@126.com.

## Author Contributions

GY and ZD: design of methodology, development and implement of models, original drafting. YZ and ZC: data curation and preprocessing. LZ, BZ, CC, and QH: experimental results analysis, draft reviewing. JT and FL: data collection. WY and XW: design of methodology, review and editing. All authors contributed to the article and approved the submitted version.

## Funding

This work was supported by the National Natural Science Foundation of China (No.82172020), the Guangzhou Science and Technology Plan (No.202102010264), and the Youth Committee of Medical Engineering Branch of Guangdong Medical Association Research Projects (No.2019-GDMAZD-01).

## Conflict of Interest

The authors declare that the research was conducted in the absence of any commercial or financial relationships that could be construed as a potential conflict of interest.

## Publisher’s Note

All claims expressed in this article are solely those of the authors and do not necessarily represent those of their affiliated organizations, or those of the publisher, the editors and the reviewers. Any product that may be evaluated in this article, or claim that may be made by its manufacturer, is not guaranteed or endorsed by the publisher.
