# Supplementary material for: Multiscale Local Enhancement Deep Convolutional Networks for the Automated 3D Segmentation of Gross Tumor Volumes in Nasopharyngeal Carcinoma: A Multi-Institutional Dataset Study
Source: Front Oncol. 2022 Mar 18;12:827991. doi: 10.3389/fonc.2022.827991 (PMC8979212; doi:10.3389/fonc.2022.827991)
Supplement: Supplementary file 1 [file Table_1.docx]

**Supplementary table 1.** The segmentation results of the models pretrained, fine-tuned and validated on the dataset A, B, C and B+C. Two-tailed p-values <0.05 were considered statistically significant.

| Training datasets | Test datasets | DSC (%) | ASSD (mm) |
| --- | --- | --- | --- |
| B (80%) | B (20%) | 72.84±9.07 | 1.89±0.88 |
|  | A | 61.23±10.77 | 2.80±2.02 |
| C (80%) | C (20%) | 65.77±8.77 | 2.56±1.58 |
|  | A | 58.88±10.59 | 2.72±3.96 |
| B Pretrained + A (70%) fine-tuning | ① A (30%) | 74.81±7.15 | 1.36±0.60 |
| C Pretrained + A (70%) fine-tuning | ② A (30%) | 74.27±6.67 | 1.40±0.57 |
| (B+C) Pretrained + A (70%) fine-tuning | ③ A (30%) | 74.46±6.38 | 1.61±1.32 |
| A (70%) | ④ A (30%) | 74.46±6.37 | 1.45±0.67 |
| P-value | ④ A vs. ① A | 0.507 | 0.116 |
|  | ④ A vs. ② A | 0.672 | 0.291 |
|  | ④ A vs. ③ A | 0.995 | 0.236 |

Note: The values were represented as mean ± standard deviation. A: Our institution; B: institution B; and C: MICCAI2019.
